# Angiostatin generating capacity and anti-tumour effects of D-penicillamine and plasminogen activators

**DOI:** 10.1186/1471-2407-6-149

**Published:** 2006-06-05

**Authors:** Renate RJ de Groot-Besseling, Theo JM Ruers, Iris L Lamers-Elemans, Cathy N Maass, Robert MW de Waal, Johan R Westphal

**Affiliations:** 1 Departments of Pathology, University Medical Centre Nijmegen, P.O. Box 9101, 6500 HB Nijmegen, The Netherlands; 2 Department of Surgery, University Medical Centre Nijmegen, P.O. Box 9101, 6500 HB Nijmegen, The Netherlands; 3 Central Animal Facility, University of Nijmegen, Geert Grooteplein 29, 6525 EZ Nijmegen, The Netherlands

## Abstract

**Background:**

Upregulation of endogenous angiostatin levels may constitute a novel anti-angiogenic, and therefore anti-tumor therapy. *In vitro*, angiostatin generation is a two-step process, starting with the conversion of plasminogen to plasmin by plasminogen activators (PAs). Next, plasmin excises angiostatin from other plasmin molecules, a process requiring a donor of a free sulfhydryl group. In previous studies, it has been demonstrated that administration of PA in combination with the free sulfhydryl donor (FSD) agents captopril or N-acetyl cysteine, resulted in angiostatin generation, and anti-angiogenic and anti-tumour activity in murine models.

**Methods:**

In this study we have investigated the angiostatin generating capacities of several FSDs. D-penicillamine proved to be most efficient in supporting the conversion of plasminogen to angiostatin *in vitro*. Next, from the optimal concentrations of tPA and D-penicillamine *in vitro*, equivalent dosages were administered to healthy Balb/c mice to explore upregulation of circulating angiostatin levels. Finally, anti-tumor effects of treatment with tPA and D-penicillamine were determined in a human melanoma xenograft model.

**Results:**

Surprisingly, we found that despite the superior angiostatin generating capacity of D-penicillamine *in vitro*, both *in vivo *angiostatin generation and anti-tumour effects of tPA/D-penicillamine treatment were impaired compared to our previous studies with tPA and captopril.

**Conclusion:**

Our results indicate that selecting the most appropriate free sulfhydryl donor for anti-angiogenic therapy in a (pre)clinical setting should be performed by *in vivo *rather than by *in vitro *studies. We conclude that D-penicillamine is not suitable for this type of therapy.

## Background

Angiogenesis is an essential step in both tumour growth and the formation of distant metastases. Therefore, inhibition of tumour angiogenesis is currently being explored as a novel anti-tumour treatment [1-5].

One class of anti-angiogenic compounds consists of proteolytic fragments of larger parent molecules. Examples of this type of angiogenesis inhibitors are angiostatin and endostatin, that are fragments of plasminogen and collagen XVIII, respectively [6,7]

Angiostatin has been shown to possess anti-angiogenic effects both *in vitro *and *in vivo*. *In vitro*, angiostatin inhibited endothelial cell migration, proliferation and tube formation [8-13]. *In vivo*, administration of angiostatin – either alone, or in combination with radiotherapy or chemotherapy – resulted in potent inhibition of tumour growth and metastasis formation in several animal tumour models [14,14-19].

Application of naturally occurring anti-angiogenic molecules in a clinical setting may be especially attractive, as these compounds have been shown to combine limited toxicity with the absence of development of drug resistance in several animal tumour models [20,16]. The step from animal model to clinical application, however, has in the case of angiostatin been hampered by problems associated with producing sufficient amounts of biologically active recombinant protein. In addition, several different angiostatin moieties have been described, possibly with different clinical efficacies [9,21,22]. As angiostatin is also present in the circulation of healthy individuals [11]; Westphal, manuscript in preparation], boosting the angiostatin generating capacities of the host might provide an alternative for exogenous angiostatin administration.

*In vitro*, angiostatin is generated via a two-step process [23]. First, plasminogen activators (PAs) convert plasminogen into the active protease plasmin. Next, plasmin excises angiostatin from other plasmin molecules or from plasminogen, in a process requiring a donor of a free sulfhydryl (-SH) group (FSD). In several recent studies, administration of tissue type plasminogen activator (tPA) and/or a FSD has actually been shown to result in anti-angiogenic activity *in vitro *and additional anti-tumour activity in murine models. Merchan et al. reported that human plasma treated with tPA and captopril displayed anti-angiogenic activity in an *in vitro *tube formation assay. *In vivo*, tPA/captopril treatment inhibited angiogenesis in Matrigel plugs. In addition, anti-angiogenic activity was present in serum from human cancer patients treated with tPA and captopril. Remarkably, the anti-angiogenic activity present in the plasma samples was not completely attributable to angiostatin, as plasma, from which angiostatin was depleted, retained its anti-angiogenic properties [24]. Agarwal et al [25] showed that administration of the FSD N-acetyl-cysteine to MDA-MB-435 breast carcinoma xenograft bearing mice resulted in angiostatin accumulation in the tumour, the induction of apoptosis in endothelial cells, a reduction of vascular density in the core of the tumour and, as a consequence, a reduced tumour growth rate. Studies performed in our laboratory have demonstrated that administration of tPA in combination with captopril to mice resulted in an increased level of circulating angiostatin. In addition, this treatment resulted in anti-tumor activity in a human melanoma xenograft model [26]. Finally, Soff reported potent anti-tumour activity in human cancer patients after treatment with uPA and captopril [27].

However, several questions remain to be addressed. Most FSDs have other characteristics beside their capability to donate a SH group that may interfere with their application in the clinical setting. Moreover, these properties may influence, either positively or negatively, their angiostatin generating capacity. Captopril, for instance, is a potent ACE inhibitor, and administration of captopril lowers the blood pressure. Impaired blood flow towards and in the tumour may have contributed to the observed reduction in tumour growth. In addition, captopril inhibits matrix metalloproteinases [28,29], molecules that are prominently involved in angiogenesis [30].

N-acetyl-cysteine (NAC), a glutathione precursor and FSD used in the study by Agarwal et al [25], not only affects angiostatin formation, but also possesses antioxidant activity, and inhibits MMPs [31]. Clearly, there is a need for preclinical studies to determine the net effects of the FSDs, and to select the FSD that is best suited for clinical studies.

In this paper we have compared the *in vitro *angiostatin generating capacities of a panel of FSDs. In our hands, D-penicillamine, a structural analog of cysteine, displayed a higher angiostatin generating capacity on a molar basis than either captopril, NAC, or other FSDs. Next, we monitored circulating angiostatin levels in mice treated with D-penicillamine and tPA, and, finally, we assessed the anti-tumour activity of the FSD/plasminogen activator treatment in an human melanoma xenograft model. Surprisingly, the superior *in vitro *angiostatin generation capacity of D-penicillamine was not reflected in either efficient *in vivo *angiostatin generation or superior anti-tumour activity. In both assays, D-penicillamine was less effective than captopril, indicating the need for further research into the most optimal free sulfhydryl group donor for clinical applications.

## Methods

### Angiostatin formation in vitro

uPA (*Sigma-Aldrich Chemie b.v., Zwijndrecht, The Netherlands*) or tPA(*Actilyse, Boehringer Ingelheim b.v., Alkmaar, The Netherlands*) and/or free sulfhydryl donor D-penicillamine, L-cysteine, N-acetyl-L-cysteine, reduced glutathione (*Sigma-Aldrich Chemie bv., Zwijndrecht, The Netherlands*) or captopril (*Bristol-Myers Squibb B.V., Woerden, The Netherlands*) were incubated with murine serum (diluted 1:40 in sterile distilled water) at 37°C for 0, 2, 4.5 or 20 hours. Different concentrations of each compound were tested. Angiostatin generation was analyzed by western blot. Conversion of plasminogen to plasmin, and generation of angiostatin were scored semi-quantitatively, as a combination of speed of formation and amount of protein formed, relative to the amount of precursor molecule as follows:

Plasminogen to plasmin:

1 No conversion at any time point

2 Slight conversion at t = 20 hours

3 Slight conversion at t = 4.5 hours

4 Moderate conversion at t = 4.5 hours

5 Moderate conversion at = 2 hours

6 Considerable conversion at t = 2 hours

7 Complete conversion at t = 2 hours

Plasmin to angiostatin:

1 No conversion

2 Slight conversion

3 Moderate conversion

4 Considerable conversion

5 (Almost) complete conversion

### Angiostatin formation *in vivo*

Healthy BALB/c mice were treated with 0.1 ml 400 mM D-penicillamine (bidaily intraperitoneal injections) and/or tPA (i.v. injection, 80 μg in 100 μl saline). Control mice received saline injections. D-penicillamine administration was started on day 0, 3 days before the first tPA injection. Mice received two tPA injections, on day 3 and on day 7. On day 7, plasma samples were collected prior to tPA injection (t = 0), and 15 minutes, 2, 4, 8, 24, or 48 hours after tPA injection. Plasminogen, plasmin and angiostatin levels in plasma were determined by western blot analysis after lysine-sepharose purification.

### Purification of angiostatin from plasma by affinity chromatography on lysine-sepharose

Lysine-sepharose (*Pharmacia Biotech, AB, Uppsala, Sweden*) was prepared according to the manufacturer's specifications. 50 μl of lysine-sepharose slurry was added to 50 μl of plasma. After incubation overnight at 4°C under continuous agitation, samples were washed three times with 50 mM phosphate buffer (pH 6.0), followed by incubation at room temperature for 30' with 50 μl 0.5 M NaCl to detach non-specifically bound proteins. Specifically bound proteins were eluted by incubation for 30' at room temperature in 50 μl 200 mM ε-aminocapronic acid. After centrifugation, supernatant was collected, mixed with an equal volume of sample buffer, and stored at -20°C prior to western blot analysis.

### Generation of rabbit anti-mouse plasminogen polyclonal antibody

Mouse plasminogen was isolated from plasma by lysine sepharose purification. A NZW rabbit was immunized with plasminogen in complete Freund's adjuvant, followed by boosters with mouse plasminogen in PBS every third week. After three boosters, plasma samples were collected and tested by western blot analysis to determine the anti-plasminogen antibody titer.

### SDS-page and western blot analysis

Western blot analysis to detect angiostatin was performed as described previously [26]. Briefly, samples were separated on 10% polyacrylamide gels under reducing conditions, and electro blotted onto Hybond-C-extra nitrocellulose membrane (*Amersham Life Science bv., Roosendaal, The Netherlands*). For analysis of plasma samples from *in vivo *experiments, a rabbit polyclonal anti-mouse plasminogen antibody was used (concentration 1: 2000 in blocking solution; our laboratory,[26]), whereas for assessment of *in vitro *angiostatin generation a polyclonal rabbit-anti-human plasminogen antibody (cross-reactive with mouse plasminogen) was used.

After Incubation with peroxidase conjugated goat-anti-rabbit antibody (Dako), blots were developed by chemoluminescence according to the manufacturer's specifications (*Roche Diagnostics GmbH*).

### Human melanoma xenograft experiment

Cell line BLM [van Muijen et al., 1991]. was cultured in Dulbecco's Modified Eagle Medium (DMEM) supplemented with glutamine (*Biowhittaker, Walkersville, MD, USA*), 10% fetal calf serum (*Integro, Zaandam, The Netherlands*) and antibiotics in plastic culture flasks (*Costar, Roskilde, Denmark*). For inoculation, cultured cells were detached from the culture flask by incubation with 0.05% trypsin/0,02% EDTA/PBS, washed, and resuspended in PBS at a concentration of 10 * 10^6 ^cells/ml. 1 * 10^6 ^Cells were injected s.c. in the flank of male 8–10 weeks-old BALB/c nu/nu mice in a volume of 100 μl. Mice were kept under aseptic conditions.

D-penicillamine administration (0.1 ml of a 400 mM solution, twice daily i.p. injection, controls received saline) was started immediately after tumor take as determined by palpation of the inoculation site. When tumours had reached a volume of 100 mm^3 ^(as determined by length × width × height measurements), intravenous tPA injections (0, or 80 μg in 100 μl saline) were administered 3 times a week, at which time points tumour volumes were determined as well. Animals were sacrificed when tumours reached a size of 1000 mm^3^. Tumours were removed and stored in liquid nitrogen. In each experiment, at least 5 mice per treatment modality were evaluated. Experiments were repeated three times.

### Immunohistochemical staining of blood vessels in human melanoma xenografts

Blood vessels were stained immunohistochemically as described previously (ref Westphal) Briefly, aceton fixed 4 μm cryo tissue sections were incubated overnight at 4°C with rat-anti-mouse endothelial cell antibody 9F1 (A generous gift from Dr. A. Hamann, Hamburg, Germany). Biotinylated rabbit-anti-rat antibody (*Vector Laboratories, Burlingame, CA*) followed by avidin-biotin-peroxidase solution and AEC (*Scytek Laboratories, Utah, USA*) were used for detection and color development.

### Counting of tumour blood vessels

The number of 9F1 positive blood vessels in melanoma xenografts was counted in three high-powered fields (magnification 200×) by two independent researchers who were blinded for treatment modality as described previously [32].

### Statistical evaluation

Statistical significance was assessed using an one-way ANOVA. Probability values of 0.05 or less were considered significant.

## Results

### Angiostatin generation *in vitro*

**Figure 1:**
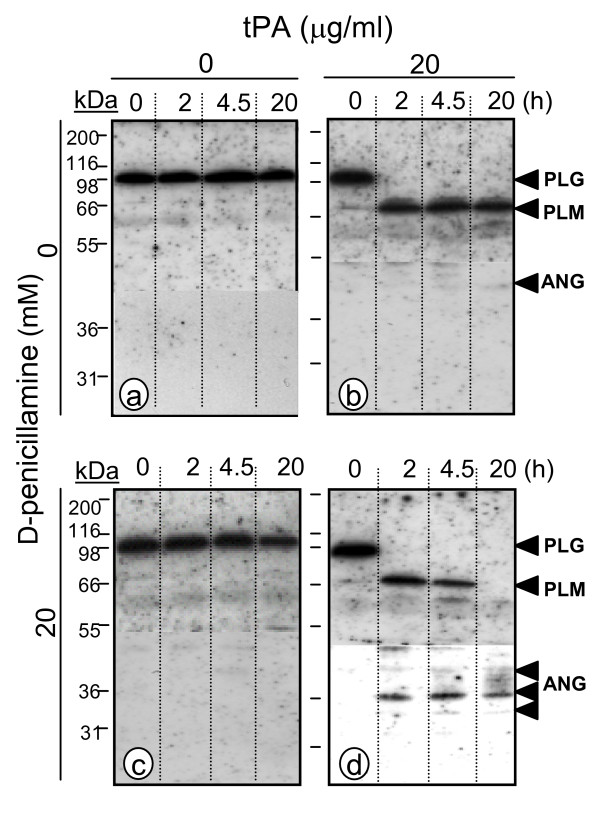
***In vitro *angiostatin generation by tPA and D-penicillamine**. Murine serum was incubated with tPA, D-penicillamine, or a combination of the two. Reaction products were subjected to Western blot analysis employing an anti-mouse plasminogen antibody. In control samples (a) or samples incubated with D-penicillamine (c), no conversion of plasminogen could be demonstrated. Incubation of serum with tPA resulted in the formation of plasmin and a small amount of angiostatin (b), whereas substantial amounts of angiostatin were observed after incubating serum with both tPA and D-penicillamine (d). PLG: plasminogen PLM: plasmine ANG: angiostatin

The optimal concentrations for *in vitro *angiostatin formation were investigated by incubating murine serum with uPA, tPA, and/or a free sulfhydryl group donor, followed by western blot analysis of the reaction products. Figure 1 illustrates a typical experiment, showing conversion of plasminogen to plasmin and angiostatin by tPA and D-penicillamine. Incubation of serum with D-penicillamine alone did not result in any plasmin or angiostatin formation (figure 1c), whereas incubation with only tPA resulted in rapid and complete conversion of plasminogen to plasmin, and the formation of a small amount of angiostatin (figure 1b). Incubation of murine serum with the combination of D-penicillamine and tPA resulted in time-dependent formation of considerable amounts of angiostatin of approximately 36 kDa, and smaller amounts of angiostatin moieties of 34 and 48 kDa (fig. 1d).

**Figure 2:**
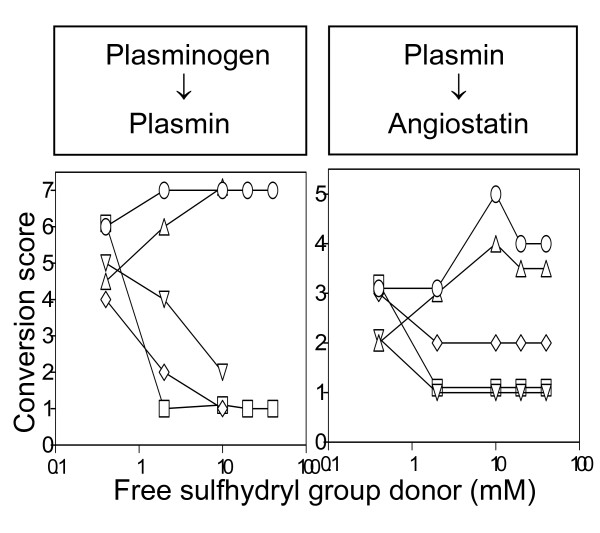
**Example of *in vitro *angiostatin generation by tissue-type plasminogen activator and FSDs**. A mixture of murine serum, tPA (2 μg/ml) and a FSD (D-penicillamine, L-cysteine, N-acetyl-L-cysteine (NAC), reduced glutathione, or captopril) was incubated for 0, 2, 4.5 or 20 hours. Conversion of plasminogen to plasmin (left panel) and plasmin to angiostatin (right panel) were scored separately on a semi-quantitative scale. D-penicillamine was the most effective FSD for generation of plasmin and angiostatin. : D-penicillamine;  : L-cysteine;  : N-acetyl L-ysteine;  : reduced glutathione;  : captopril.

In figure 2 an example of *in vitro *plasmin and angiostatin generation by tPA (2 μg/ml) and the different FSDs is demonstrated. Conversion of plasminogen to plasmin (left panel) and plasmin to angiostatin (right panel) were scored separately on a semi-quantitative scale. Compared to the other FSDs D-penicillamine induced the rapid and effective plasmin and angiostatin generation.

**Figure 3:**
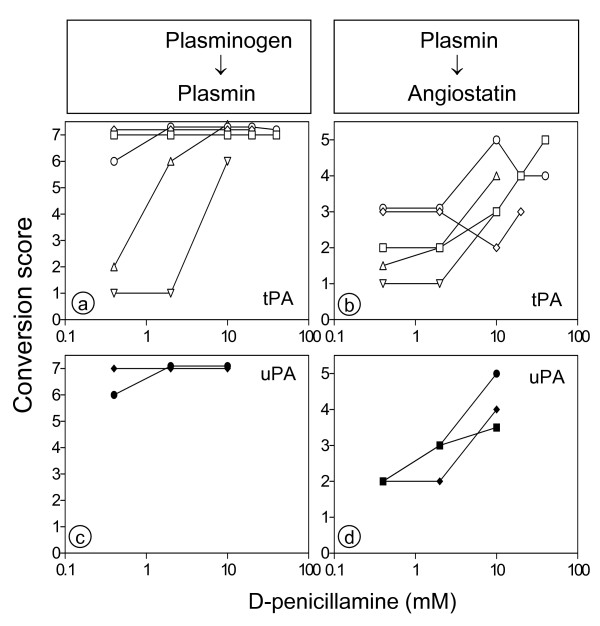
**Summary of *in vitro *angiostatin generation by plasminogen activators and D-penicillamine**. Murine serum was incubated for 0, 2, 4.5 or 20 hours with D-penicillamine and tPA (a and b) or uPA (c and d). Conversion of plasminogen to plasmin (a and c) and plasmin to angiostatin (b and d) were scored separately on a semi-quantitative scale. Note concentration dependent conversion of plasminogen to plasmin (a), and conversion of plasmin to angiostatin (b) with increasing concentrations of tPA and D-penicillamine (b). Surprisingly, uPA was very efficient in plasmin generation (c), while with increasing uPA concentrations a reciprocal effect on angiostatin formation was observed (d). tPA concentrations (μg/ml): : 0.08;  0.4; : 2;  : 4;  : 20 and 100 uPA concentrations (units/ml):  : 0.004;  : 0.02;  : 0.1.

Figure 3 shows a summary of experiments performed to determine the optimal plasminogen activator (figure 3a and 3b: tPA, figure 3c and 3d: uPA) and D-penicillamine concentrations for *in vitro *angiostatin generation. Conversion of plasminogen to plasmin (figure 3a and 3c) and plasmin to angiostatin (figure 3b and 3d) were scored as described above.

D-penicillamine and tPA induced conversion of plasminogen to plasmin and plasmin to angiostatin showed a clear dose-dependency. 2 mM D-penicillamine combined with 2 μg/ml tPA or more resulted in complete conversion of plasminogen to plasmin after 2 hours of incubation. Angiostatin formation was optimal at 4 μg/ml tPA and 20 mM D-penicillamine.

Plasmin generation by uPA in the presence of D-penicillamine was already complete at low uPA (0.02 unit/ml) and D-penicillamine (0.2 mM) concentrations. Remarkably, angiostatin formation induced by uPA and D-penicillamine showed an inverse dose-response relationship with regard to the amount of uPA. Whereas higher D-penicillamine concentrations resulted in the formation of more angiostatin, increased levels of uPA resulted in less angiostatin formation.

Since angiostatin formation by uPA and D-penicillamine was subject to inhibition by higher uPA concentrations, all subsequent *in vivo *experiments were conducted with tPA and D-penicillamine.

### Angiostatin generation *in vivo*

**Figure 4:**
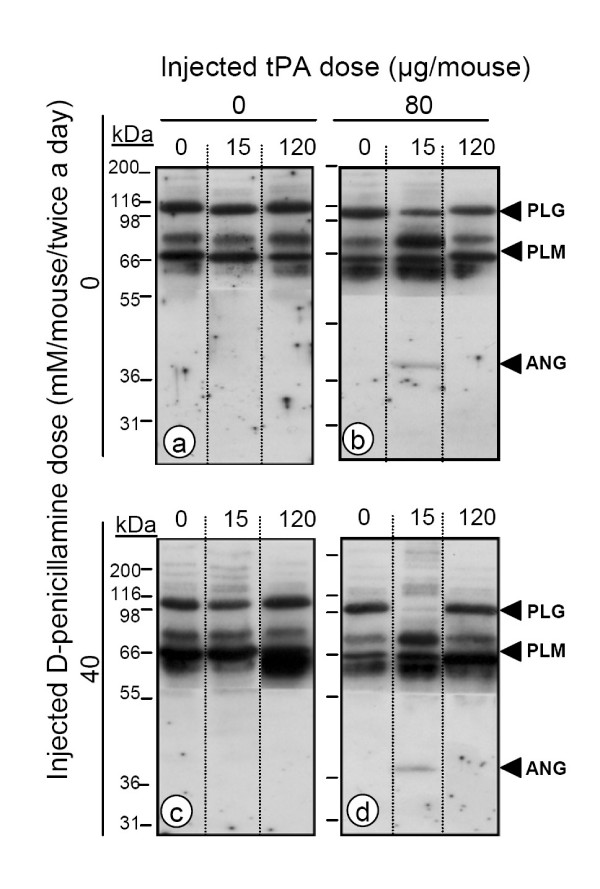
***In vivo *angiostatin formation in healthy mice after treatment with tPA and/or D-penicillamine**. *In vivo *angiostatin generation was induced by administration of tPA (two i.v. injections, on day 3 and 7) and/or D-penicillamine (i.p. injection, twice daily, starting prior to the first tPA injection) to healthy BALB/c mice. Plasma samples were collected prior to the tPA injection on day 7 (t = 0), and 15 or 120 minutes after tPA injection. After purification by lysine sepharose affinity chromatography, samples were analyzed on western blot. Note a complete conversion of plasminogen to plasmin, and the formation of angiostatin in mice receiving tPA and D-penicillamine, 15 minutes after tPA injection.

To determine whether we could induce angiostatin formation *in vivo*, either D-penicillamine (0.1 ml of a 400 mM solution, bidaily i.p. injections prior to tPA injection), or tPA (two i.v. injections, 3 days and 7 days after start of the D-penicillamine/saline injections), or a combination of the two, were administered to healthy BALB/c mice. After 7 days, plasma samples were collected prior to the tPA injection (t = 0), and 15 and 120 minutes after tPA injection. Plasminogen, plasmin, and angiostatin levels were analyzed by western blot (figure 4).

In plasma from mice receiving only saline injections (figure 4a) or D-penicillamine (figure 4c), both plasminogen (110 kDa) and plasmin heavy chain (approximately 70 kDa) were observed in the circulation. No detectable levels of angiostatin were present. In mice receiving only tPA (figure 4b), an incomplete conversion of plasminogen to plasmin was observed 15 minutes after tPA injection. After two hours the levels of plasminogen and plasmin had returned to normal. The generation of an angiostatin band at 36 kDa was observed 15 minutes after the tPA injection. 120 Minutes after tPA injection, no angiostatin was detectable anymore.

Administration of both tPA and D-penicillamine resulted in a complete conversion of plasminogen to plasmin, and formation of angiostatin 15 minutes after tPA injection. Remarkably, the increased conversion of plasminogen to plasmin did not result in higher levels of circulating angiostatin compared to injection with tPA alone (figure 4b vs. 4d). Both plasminogen and angiostatin levels had returned to normal two hours after tPA injection.

### Anti-tumour effect of D-penicillamine/tPA treatment

**Figure 5:**
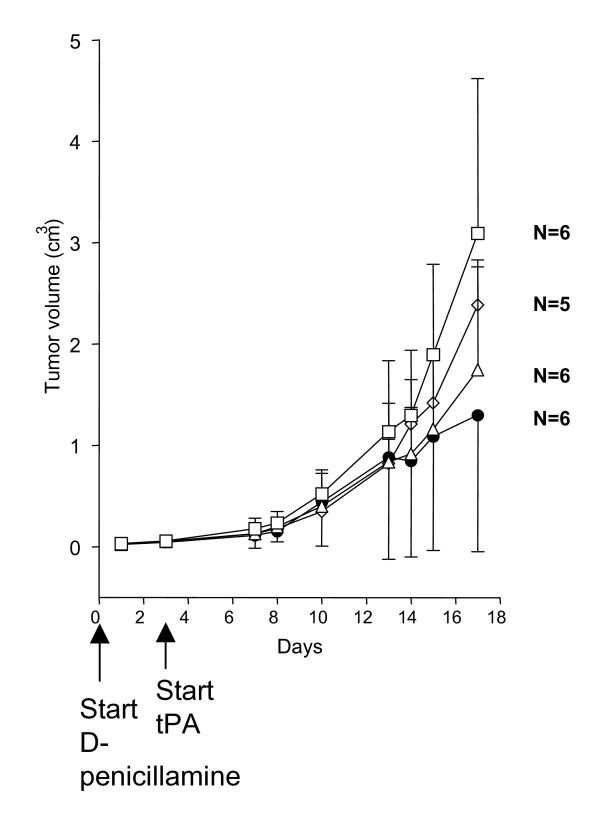
**Anti-tumor effects of tPA and D-penicillamine in a human melanoma xenograft model**. Nude mice xenografted with human melanoma BLM were treated with tPA, D-penicillamine, or a combination of tPA and D-penicillamine. Shown are mean tumor volumes of at least 5 mice/treatment modality plus/minus SD. A reduction in tumor growth of 58% was observed by applying the combination treatment schedule. Treatment schedules:  : none;  : D-penicillamine;  : tPA; : tPA and D-penicillamine

The anti-tumour effect of D-penicillamine/tPA treatment was assessed in a human melanoma xenograft model. After s.c. injection of human BLM melanoma cells in the flank, nude mice were treated with D-penicillamine or saline injections (i.p. injections, twice daily when tumour take was observed. The tPA or saline injections (i.v. three times a week) started when tumours reached a size of 100 mm^3^. Tumour volumes were measured to monitor the effect of the treatment. Figure 5 shows a representative example of three different experiments.

In mice receiving only D-penicillamine injections, a small reduction in tumour growth was observed (23% inhibition, 17 days after start of treatment), whereas tPA alone resulted in a 44% reduction in tumour growth. The combination of D-penicillamine and tPA inhibited tumor growth by 58%. The observed inhibition of tumour growth, however, was not statistically significant (p = 0.08). In contrast to healthy mice, angiostatin was not detectable by western blot analysis of plasma samples collected from xenograft-bearing mice treated with tPA and/or D-penicillamine (data not shown).

### Vascular densities of human melanoma xenografts

**Figure 6:**
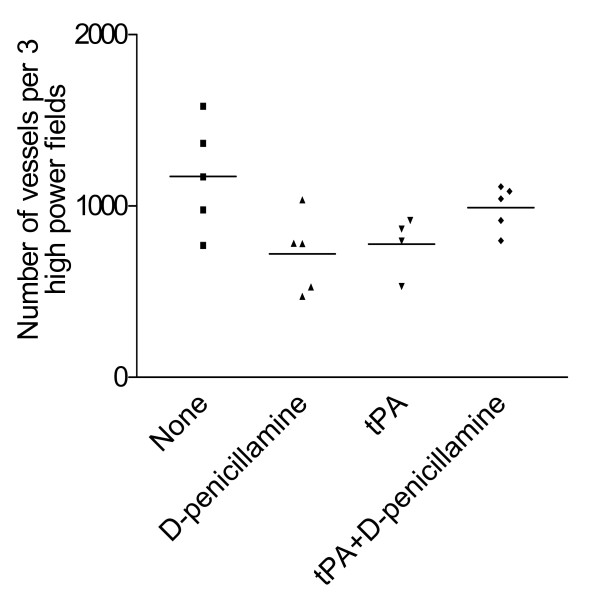
**Treatment effects on the amount of tumor vessels**. In tissue sections from BLM tumors derived from mice treated with tPA and/or D-penicillamine, blood vessels were stained with rat-anti-mouse-endothelial cell antibody 9F1, and quantified. Although a reduction in vessel density was observed after either treatment, these reductions did not reach statistical significance.

To determine a possible anti-angiogenic effect of the tPA/D-penicillamine treatment, blood vessels in melanoma xenografts were stained with rat-anti-mouse endothelial cell antibody 9F1 and counted in three high-powered microscopic fields (figure 6).

The mean number of blood vessels decreased by 34 and 39% in tumours derived from mice treated with only tPA or D-penicillamine, respectively, compared to tumours from control mice. Remarkably, the reduction in vessel density was less in tumours from mice that had been received the combination treatment (16% compared to control tumours). All observed reductions in vascular density were not statistically significant.

## Discussion

In a series of recent publications, the positive effect of a combination of a plasminogen activator (PA) and a free sulfhydryl group donor (FSD) on *in vitro *and *in vivo *angiostatin generation was described. In addition, treatment with PA plus FSD, or FSD alone, displayed anti-angiogenic and anti-tumour effects in murine xenograft tumour models [24-26] and in a limited number of human patients [27]. This novel approach to anti-angiogenic therapy may utilize approved drugs such as captopril as FSD.

In this paper we set out to identify the most potent FSD with regard to angiostatin generation. In addition, testing different FSDs with different additional properties next to donating an SH group, may shed more light on the actual working mechanism of the anti-angiogenic effects induced by these drugs.

Our *in vitro *data demonstrated that, in the presence of plasminogen activators, all tested FSDs were able to induce angiostatin generation *in vitro*, in accordance with data reported by Gately et al [Gately et al., 1997]. In line with previous experiments with tPA and captopril? [26], we observed formation of angiostatin moieties with molecular weights varying between 30 and 50 kDa under reducing conditions. The 36 and 48 kDa moieties may represent the 3 and 4 kringle forms of angiostatin, respectively. A dose response curve was observed for the angiostatin formation induced by tPA and D-penicillamine. Remarkably, increasing uPA concentrations in the presence of FSDs did not result in increased angiostatin levels, but rather in an inhibition of the formation of this molecule. The reason for this phenomenon was unclear. Hypothesizing, it could be that (too) high levels of uPA, cause the activation of negative feedback mechanisms, resulting in the inactivation of plasmin, and as a consequence in a reduced generation of angiostatin. For this reason, we decided to perform our *in vivo *studies with tPA.

Surprisingly, not only angiostatin generation, but also the conversion of plasminogen to plasmin was enhanced by FSD addition, suggesting that this first conversion step is, at least in part, FSD-dependent as well. To our knowledge, this has never been observed before. In future experiments, we will test this plasmin-activating capacity of FSDs. Of all tested FSDs, D-penicillamine was, on a molar basis, most potent in inducing both plasmin and angiostatin formation. Therefore, D-penicillamine was used in all subsequent *in vivo *studies.

Although the tPA-induced conversion of plasminogen to plasmin *in vivo *was also increased by co-treatment with D-penicillamine, no increased angiostatin formation compared to treatment with tPA alone was observed. In contrast to the multiple molecular weight forms of angiostatin that we observed in our *in vitro *experiments, *in vivo *only the formation of a single angiostatin species of 36 kDa, comparable to angiostatin generated by tPA and captopril in previous studies, could be observed. Different angiostatin moieties might express different anti-angiogenic effects *in vivo*, although this is at present unclear. Thus far, only *in vitro *studies have compared the anti-angiogenic properties of plasminogen fragments containing kringles 1–3, 1–4, 1–5 and 5 [Cao et al., 2002;Lucas et al., 1998;Cao et al., 1996].

The fact that plasminogen, plasmin and angiostatin levels had returned to normal within two hours after the tPA injection, suggested a relatively short biological T_1/2 _of both tPA and angiostatin. Whether angiostatin is subject to quick renal excretion, is degraded to shorter fragments, or disappears from the circulation due to other mechanisms, cannot be concluded from our experiments.

When treating mice bearing human melanoma xenografts, administration with either tPA or D-penicillamine alone resulted in an inhibition of tumour growth (44% and 23%, respectively). Treatment with both drugs resulted in a cumulative inhibition (58%). It should be noted, however, that the observed inhibition of tumour growth was not statistically significant, and therefore was far from convincing. In accordance with this finding, mean vascular densities in treated tumours where somewhat lower than in untreated tumours, but again not statistically significant. Matsubara et al (1989) [33] found that D-penicillamine only reduced endothelial cell proliferation in the presence of copper sulfate. Treatment of D-penicillamine alone did not alter the proliferation. The lack of administration of copper sulfate during our experiments might be the cause of the somewhat disappointing in vivo effects. However, Matsubara et al (1989) [33] also treated rabbits with D-penicilamine, and these experiments did result into inhibition of vessels growth in the cornea. But their administration route was intravenously, and not intraperitoneal as our route was. This could made the differences in the anti-angiogenic effects of D-penicillamine observed in both experiments.

In contrast to our D-penicillamine data, co-administration of captopril to mice treated with tPA in similar experiments, resulted in a clearly enhanced formation of angiostatin, and in a significant inhibition of tumour growth [26]. We therefore concluded that the superior capacity of D-penicillamine to induce angiostatin formation *in vitro *compared to captopril and other FSDs, was antagonized by environmental factors present in the living organism, resulting in a failure to significantly inhibit tumour growth.

Several explanations for this finding can be offered. Both captopril and D-penicillamine possess other characteristics that may influence angiogenesis and tumour growth.

First, as discussed previously, the drop in blood pressure induced by captopril-mediated ACE inhibition may account for some of its anti-tumour activity. D-penicillamine does not alter blood pressure.

Second, captopril may inhibit MMP function, thus preventing angiogenesis. D-penicillamine, on the other hand, does not affect blood pressure, but might share with captopril the ability to inhibit MMPs, by chelating metal ions. MMPs require zinc to express their activity. Therefore, chelation of these zinc molecules by D-penicillamine prevents the activation of MMPs. Moreover, it is known that D-penicillamine suppresses the inhibitory effects of hypochlorous acid (HOCl) on TIMP-1 (tissue inhibitor of metalloproteinase-1). As a consequence, TIMP-1 is not inhibited anymore, and could express its activity: limiting MMP activity [34].

Third, the fact that D-penicillamine binds with high affinity to albumin [35,36] migt be of importance. By binding to albumin, the bioavailability, and therefore efficacy, of free circulating D-penicillamine is reduced. D-penicillamine sequestered to albumin may be less capable, or even unable, to donate its SH groups.

The fact that we could not detect angiostatin in tumour-bearing mice treated with tPA or tPA plus D-penicillamine suggested that circulating angiostatin is taken up by the tumour, for instance by binding of angiostatin to tumour blood vessels. As, however, immunohistochemical staining for angiostatin requires a specific antibody that does not stain the precursor (plasminogen) and this is not available, this could not be confirmed.

Another explanation for the absence of angiostatin could be that other anti-angiogenic factors might be present in our model. In our studies with tPA and captopril [26], and D-penicillamine (this paper), as well as in the study published by Agarwal with N-acetyl-cysteine (NAC), it is unclear whether angiostatin was in fact the effector molecule responsible for the observed anti-angiogenic and anti-tumour effects. Although Agarwal described accumulation of angiostatin in tumors derived from mice treated with NAC, no depletion studies were performed.

Agarwal et al. only administered the FSD N-acetyl-cysteine to achieve anti-angiogenic and anti-tumour activity, without the need for additional plasminogen activator administration. *In vitro *it was shown that tumour-derived VEGF induced expression of uPA and uPAR on HUVEC. In addition, uPA and uPAR expression was also demonstrated on tumour endothelium of NAC-treated mice. Apparently, this endothelium-derived uPA was sufficient to produce angiostatin. This notion is supported by our observations that both captopril and D-penicillamine are able to induce some (although not significant) inhibition of tumour growth. Additionally, captopril alone has been shown to possess anti-tumour activity in a human Karposi sarcoma patient [37], and in murine tumour models [38,28,29].

Furthermore, plasminogen activators expressed by the tumours used in these experiments, may have contributed to the observed anti-tumour effects after treatment with captopril or D-penicillamine alone. The human melanoma cell line BLM we used in our xenograft growth inhibition experiments expresses high levels of tPA. In both cases, however, administration of exogenous tPA improved anti-tumour activity, suggesting that the efficacy of the N-acetyl-cysteine treatment as reported by Agarwal may benefit from administration of exogenous plasminogen activators as well.

## Conclusion

In conclusion, recent studies have provided evidence that upregulation of endogenous anti-angiogenic molecules as a novel anti-tumour therapy is feasible. Further studies are required to identify the most efficient plasminogen activators and FSDs, their optimal concentrations, and the appropriate application strategies. Regarding the dramatically different angiostatin generating capacities of D-penicillamine *in vitro *versus *in vivo*, our results indicate that selecting the most appropriate free sulfhydryl donor for anti-angiogenic therapy in a (pre)clinical setting should be performed by *in vivo *rather than by *in vitro *studies. Finally, we conclude that D-penicillamine is not suitable for this type of therapy.

## Competing interests

The author(s) declare that they have no competing interests.

## Authors' contributions

RRJdGB: substantial contributions to the conception and design, to the acquisition of data, and to the analysis and interpretation of data, involved in drafting the manuscript and revising it critically, have given final approval of the version to be published.

ILLE: substantial contributions to the conception and design, to the acquisition of data, have given final approval of the version to be published.

CNM: substantial contributions to the conception and design, to the acquisition of data, have given final approval of the version to be published.

RMWdW: substantial contributions to the conception and design, to the analysis and interpretation of data, involved in revising the manuscript critically, have given final approval of the version to be published.

TJMR: substantial contributions to the conception and design, to the analysis and interpretation of data, involved in revising the manuscript critically, have given final approval of the version to be published.

JRW: substantial contributions to the conception and design, to the analysis and interpretation of data, involved in revising the manuscript critically, have given final approval of the version to be published.

## Pre-publication history

The pre-publication history for this paper can be accessed here:
